# Domain architecture conservation in orthologs

**DOI:** 10.1186/1471-2105-12-326

**Published:** 2011-08-05

**Authors:** Kristoffer Forslund, Isabella Pekkari, Erik LL Sonnhammer

**Affiliations:** 1 Stockholm Bioinformatics Centre, Science for Life Laboratory, Box 1031, Solna, 17121 Sweden; 2 Department of Biochemistry and Biophysics, Stockholm University; 3 Swedish eScience Research Center

## Abstract

**Background:**

As orthologous proteins are expected to retain function more often than other homologs, they are often used for functional annotation transfer between species. However, ortholog identification methods do not take into account changes in domain architecture, which are likely to modify a protein's function. By domain architecture we refer to the sequential arrangement of domains along a protein sequence.

To assess the level of domain architecture conservation among orthologs, we carried out a large-scale study of such events between human and 40 other species spanning the entire evolutionary range. We designed a score to measure domain architecture similarity and used it to analyze differences in domain architecture conservation between orthologs and paralogs relative to the conservation of primary sequence. We also statistically characterized the extents of different types of domain swapping events across pairs of orthologs and paralogs.

**Results:**

The analysis shows that orthologs exhibit greater domain architecture conservation than paralogous homologs, even when differences in average sequence divergence are compensated for, for homologs that have diverged beyond a certain threshold. We interpret this as an indication of a stronger selective pressure on orthologs than paralogs to retain the domain architecture required for the proteins to perform a specific function. In general, orthologs as well as the closest paralogous homologs have very similar domain architectures, even at large evolutionary separation.

The most common domain architecture changes observed in both ortholog and paralog pairs involved insertion/deletion of new domains, while domain shuffling and segment duplication/deletion were very infrequent.

**Conclusions:**

On the whole, our results support the hypothesis that function conservation between orthologs demands higher domain architecture conservation than other types of homologs, relative to primary sequence conservation. This supports the notion that orthologs are functionally more similar than other types of homologs at the same evolutionary distance.

## Background

Genes are homologous if they share a common evolutionary origin. Homologous genes in different species are defined as orthologs if they descend from a single gene in the last common ancestor [1], and outparalogs, if they diverged via duplication before this ancestor. For two orthologous genes, if either has duplicated since the speciation event that separated them, the resulting copies in one species will all be inparalogs with respect to each other, and co-orthologs to the corresponding gene(s) in the other species. All the genes in two species which descend from a single gene in the last common ancestor of those species form a set of genes called an ortholog cluster or group [2,3].

The more genomes are sequenced, the more important orthology identification becomes. This is because orthologs often have the same or closely related functions in the extant species and therefore can be used for the transfer of functional information. For example, the functions of human genes can be identified by studying their orthologs in other organisms. Likewise, transfer of functional information between orthologs is important for annotation of newly sequenced genomes [4,5]. However, while there is significant evidence that orthologous proteins generally have similar functions [4,6], the assumption that orthologs are functionally more conserved than other homologs at the same separation has not been systematically evaluated [7].

Protein domains are modules of protein architecture, typically made of independently folding subsequences that can be found in different arrangements in different proteins. The Pfam database [8,9] contains assignments of domains to the proteins in the major protein databases. These Pfam-A domains are made using Hidden Markov Models (HMMs) trained on manually curated seed sets of biologically relevant protein domains. We define a domain architecture as the sequential arrangement of Pfam-A domains along a protein sequence, in N- to C-terminal order.

Proteins evolve by point mutations, insertions, and deletions. The latter two can occur locally, involving a few residues, or affect one or more entire domains [10-13]. Domain-level mutations often occur through gene fusion events or because new stop codons truncate or split genes. One expects domain-level mutations to be more common towards protein termini because these are less buried and therefore more replaceable than domains towards the middle. Such a bias towards architecture changes at the termini rather than in the middle has indeed been observed in several studies [12,13]. It should, however, be noted that the frequency of terminal domain architecture change events could be overestimated in some datasets, because the risk of error occurring in gene detection is higher by the edges of proteins. Furthermore, it has been shown that the function of a protein can be affected by changes in its domain architecture [14], or predicted from its domain content [15-17].

Previous work suggests that orthologs tend to have the same domain architecture. Lin et al. [18] investigated the degree to which Pfam domain architectures are conserved within KOG [19] ortholog clusters and found that 81% of all domain architectures were found in a single KOG only, and that 65% of all KOGs included no more than one distinct domain architecture. It is thus clear that orthologs tend to have the same domain architecture. But are they more similar in this regard than paralogs at the same degree of evolutionary separation? Orthologs are generally expected to experience stronger evolutionary pressure to maintain the same function than paralogs. If the functional roles shared by orthologs are filled by means of the proteins possessing a specific domain architecture - that is, if domain architecture is an important vehicle of protein function - then we should see this greater pressure expressed through higher conservation of domain architecture. If orthologs have relatively more conserved domain architectures than paralogs at the same evolutionary distance, such conservation may also be useful to distinguish between the two relationships for specific proteins. To test the above hypothesis, we compared the degree of domain architecture conservation between pairs of orthologous and paralogous proteins relative to their evolutionary divergence.

Various measures of domain conservation have been suggested [10,18,20]. Lin et al [18] introduced a composite metric for architecture similarity with separate contributions from domain content and domain order. The parameters of this metric were optimized for resolving homologs versus non-homologs. Because of this, it is relatively complex and less ideal for applications where the primary purpose is to compare domain architectures for its own sake, rather than as an indicator of homology. The edit distance based method of Björklund et al [10] is more straightforward for this purpose, but it is a distance measure rather than a similarity measure, and also not independent of the number of domains in a protein. Because of these limitations, we constructed a metric using an edit distance-based framework similar to that of Björklund et al [10], but inverted and normalized to produce a similarity measure, with some additional steps taken to handle phenomena such as variable-length repeat/motif domain stretches.

In the present paper, pairs of orthologs from InParanoid [21] were compared to pairs of outparalogs and inparalogs with regards to domain architecture conservation, relative to the conservation of primary sequence. The human proteome and the proteome of 40 other species spanning the entire range of evolutionary distances, were considered. For each species comparison, pairs of orthologous and and non-orthologous proteins were generated. Where differences in domain architecture were found, the domain swapping events that best explained each case were inferred. We also reassessed previous findings that domain change events are more common at the ends of proteins than in the middle. To explore domain architecture conservation among co-orthologs where gene duplication has occurred following the divergence of the two species, we also performed the same analysis for pairs of inparalogs and outparalogs. This large-scale study provides solid conclusions about the effect that orthology has on domain architecture conservation.

## Methods

### Species and sequences

Proteomes were downloaded for all the species in version 6.0 of the InParanoid database http://InParanoid.sbc.su.se[22]. These are nonredundant in the sense of containing only the longest protein sequence for each gene, regardless of the existence of alternate spliceforms. Additionally, proteomes for the bacteria *Mycobacterium tuberculosis *and *Mycobacterium leprae *as well as for the archaea *Aeropyrum pernix*, *Methanococcus acetivorans*, *Pyrobaculum aerophilum *and *Sulfolobus acidocaldarius *were downloaded from the COGENT database [23]. These files were downloaded on February 5 and February 6, 2008, respectively, and the proteomes, being prokaryotic and hence lacking a splicing machinery, were assumed to be non-redundant. Protein domain architectures were assigned under version 23.0 of Pfam [9]. Additional file 1, Table S1 shows the full selection of species, as well as the number of clusters and proteins that were included in each species analysis.

### Construction of ortholog clusters

Version 4.0 of the InParanoid algorithm was used to generate ortholog clusters for the set of species included. Ortholog and inparalog pairs were defined as pairs of proteins that are part of the same cluster in the same or different species, respectively. Such clusters are generated by scoring each inparalog to the seed ortholog, but not to each other. The missing inparalog-inparalog scores therefore had to be generated. For 547 out of 3497058 (0.016%) protein pairs considered, BLAST failed to align and score an inparalog pair in one or both directions; these pairs were excluded from the analysis.

### Identification of closest outparalogs

For all ortholog clusters in each species comparison made in this study, we identified the closest outparalogous proteins in the same and the other species. The former was defined as the sequence for which the BLAST bit score was highest out of all sequences in the same species that were not in the same ortholog cluster as A. The latter was defined similarly as the highest-scoring of all sequences from the other species that fall outside the ortholog cluster. The BLAST files generated when running InParanoid were used for the pairwise sequence scores. InParanoid default parameters were used: a score cut-off of 40 bits, an overlap cut-off of 50%, and a segment cut-off of 25%. If no significant matches remained after the removal of sequences clustered in the same cluster, the sequence was considered to lack a closest outparalog in that comparison.

### Pfam domain architectures

In this work, the term "domain architecture" (sometimes referred to as domain arrangement or domain order) specifically means the sequential arrangement of known Pfam-A domains along a protein, in N- to C-terminal order. Pfam-A protein domain architectures were determined using the HMMER [24] software under version 23.0 of Pfam. Where proteins contained several consecutive domains of type Motif or Repeat, these were collapsed into a single pseudo-domain, following the approach used previously [25]. Differences in repeat number of this type are considered evolutionarily and functionally less relevant because of how easily the number of repeats change relative to other domain architecture changes. Stretches of consecutive Pfam-A domains of type Family or Domain were retained without collapsing. Pfam domains belonging to the same clan were considered equivalent with regards to the degree of domain architecture conservation. The analysis was limited to proteins with at least one Pfam-A domain assigned.

### Domain architecture comparisons

Domain architectures were compared by using pairwise domain architecture alignments. Four different protein pair categories were analyzed. Ortholog-ortholog (O) pairs are comparisons of proteins, one from each species, which are part of the same cluster. Inparalog-inparalog (iP) pairs are comparisons of proteins from the same species that are part of the same cluster. Closest cross-species outparalog (oPx) pairs are comparisons of proteins, one from each species, where one of the proteins is part of a given orthology cluster, and the other is the closest protein in that species which is not part of the cluster. Closest same-species outparalog (oPs) pairs are comparisons of proteins from the same species, one of them part of a given cluster, and the other the closest same-species protein which is not part of the cluster.

### Pairwise domain architecture alignment creation

We used a variant of the Needleman-Wunsch algorithm [26] to align the Pfam-A domain architectures of proteins. A domain match was given a score of 0, a gap was given a score of 3, and a mismatch was assigned a score of 10. The high mismatch score was used to avoid aligning different domains with each other; that is, there is no biological reason for this specific choice of scores. Affine gap penalties were not used.

### Scoring of domain architecture alignments

For each alignment, a Domain Architecture similarity score (DA-score) was computed. This is based on the DCS (Domain Content Similarity) score described by Song et al [20] and adapted to take domain numbers and positions into account. The DA-score is defined as follows:

(1) $$DA-score\left(p_{1},p_{2}\right)=\frac{a_{12}}{\left(n_{1}+n_{2}\right)}$$

where n_1 _is the number of domains in protein p_1_, n_2 _is the number of domains in protein p_2 _and a_12 _is the number of domains in p_1 _and p_2 _which are aligned against an identical domain or a domain from the same Pfam clan. Another way of scoring domain architecture similarity in this framework would have been to use the Needleman-Wunsch score of the optimal alignment. We decided against this for several reasons. First, the impact on the result of the scoring scheme parameters would be larger, meaning that the already difficult problem of finding a biologically sensible scoring scheme would become more acute. Assuming that our present scheme produces sensible alignments, the above score is insensitive to our choice of scoring scheme parameters. Second, the DA-score as defined above has the useful property that it ranges from 0 for proteins with disjoint domain sets to 1 for proteins with identical architectures.

A total DA-score representing all alignments and clusters in a comparison was calculated. Some clusters are large and therefore generate large numbers of alignments when all-versus-all ortholog or inparalog pair comparisons are made. Averaging of scores for each cluster was done to ensure equal weight for each cluster. Total scores were calculated by averaging the mean scores for all clusters. The final similarity scores are therefore means of mean scores. Each protein pair will contribute only once, with one exception: where two proteins in different clusters are both each other's closest non-clustermate homolog, that pair will contribute to the mean score of both clusters.

### Protein sequence divergence

Sequence identity across all pairs of proteins was computed by aligning the two proteins against each other using version 2.04 of KALIGN [27], with default settings. The sequence identity was taken as the ratio of identical residues to aligned residues, and the p-distance *d *equivalently becomes the ratio of residue differences to aligned residues. For the analysis of the sequence divergence dependence of the DA-score, the p-distances were converted into evolutionary distances using the Jukes-Cantor correction for amino acid sequences [28],

(2) $$d_{JC}=-\left(\frac{19}{20}\right)\text{ln}\left(\text{1}-\frac{20}{19}d\right).$$

### Annotation of pairwise domain architecture alignments

**Figure 1:**
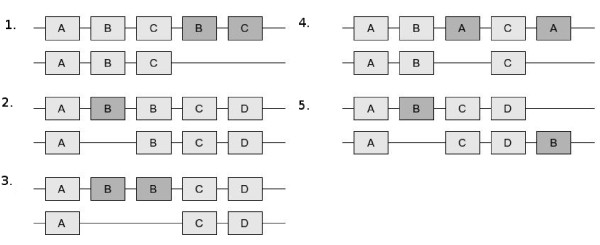
**Annotations of domain swapping events**. Aligned domains are shaded in light gray while unaligned domains are shaded in dark gray. All cases of unaligned domains are given one of five annotations. 1) Segment duplication/deletion. A segment of two or more domains were duplicated adjacently, or was lost. 2) Repetition difference. The first protein has one more B domain than the other protein. Since the unaligned domain is located next to an aligned domain of the same type, the unaligned domain is annotated as a repetition difference. 3) Insertion/deletion of a new domain. The first protein has two unaligned B domains. Both of these domains will be annotated as insertion/deletion of a new domain, since the other protein lacks domains of that type. 4) Insertion/deletion of an existing domain. The first protein has two unaligned A domains. Since the other protein has a domain of that type, the unaligned domains in the first protein will be annotated as insertion/deletion of an existing domain. 5) Domain shuffling. Both proteins have unaligned B domains. None of these domains occurs next to an aligned B domain and therefore they cannot be annotated as repetition differences. These domains will instead be annotated as exhibiting domain shuffling.

All alignments that were not classified as identical or wholly dissimilar were annotated further. All differences between the aligned domain architectures were annotated as repetition differences, insertions/deletions of new domains, insertions/deletions of existing domains, segment duplications/deletions or domain shufflings. An unaligned domain could not have more than one annotation and all unaligned domains were given one of the five different annotations. Figure 1 shows situations where the five different annotations are used. As previously stated, all domains belonging to the same Pfam clan were considered identical.

A segment duplication/deletion (the two cases cannot be distinguished between in the context of this method) is defined as an unaligned segment of domains that is placed next to an identical aligned segment. A segment must be at least two domains long and must consist of domains of at least two different types. The unaligned segment can be longer than the aligned segment, but must then consist of repeats of the aligned segment.

Assigning domain swapping event classes to protein pairs in order to explain their architectural differences was done by testing for the different events 1-5, in that order of priority (see Figure 1). There is thus an implicit precedence order when multiple possible events could explain the differences. The precedence order chosen here, while ad hoc, was considered to be reasonable with regards to how difficult each type of event might be for evolution to accomplish. In particular, changes in domain content were considered more uncommon than mere changes in order.

### Classifying the position of a domain architecture change event

For two aligned architectures, a difference between them, which was considered an architecture change event, was classified as being in the middle of the protein if there were aligned (unchanged) domains both N-terminal and C-terminal to it. If there were aligned domains only N-terminal to the change event, it was classified as a C-terminal change, and if there were aligned domains only C-terminal to the change event, it was classified as an N-terminal change.

### Normalization by cluster size

The sizes of ortholog clusters vary extensively, either due to lineage-specific expansion of gene families or because of multiple splice forms sometimes being included. This variation could potentially bias the analysis in favor of a subset of proteins, particularly for analysis of inparalog-inparalog pairs. Consequently, in the analyses that involved averaging over clusters, as well as in the analysis of the positional bias of architecture changes, we computed the average similarity of the pairs formed by each cluster, then the average similarity of these scores across all clusters.

### Binning analysis

The protein pairs from each category were divided up into bins based on Jukes-Cantor corrected amino acid distance. The thresholds for each bin were selected individually for each comparison of two pair types using the following procedure. Pairs in each category were sorted on the binning variable, and, separately for each category, divided into N consecutive bins containing an equal number of points. The binning variable thresholds corresponding to these bins were then averaged across the two categories. Sometimes, this left certain bins with too few protein pairs in either category. Because of this, bin sizes were iteratively refined. First, the bin and category that contained the smallest number of pairs was identified. Each bin has a lower and upper threshold (one in the case of the edge bins). The distance these thresholds must shift to make the number of pairs of this category in the bin the same as in the neighbor bins was estimated, approximating each bin as having uniform pair density. If performing this shift in bin thresholds increased the size of the then-smallest bin × category combination, the change was accepted and another iteration started. If it was rejected, or if the smallest bin and category contained at least one percent of the total number of pairs in the dataset as a whole, the bin threshold set as a whole was accepted and the iteration procedure stopped. In some rare cases when dividing into N = 100 bins, this algorithm may yield a bin for which one category contains no points. In this case, no analysis is performed for that particular bin.

### Statistical significance tests

To test whether pairs from two categories (such as O vs oPx) differed significantly with respect to mean DA-score within a bin, we performed a randomized permutation test [29]. The protein pairs from both categories were pooled, and new sets of protein pairs, equal in size to the two categories, were drawn from this pool. This process was repeated 1000 times, and the number of times that the difference in mean between the two sampled sets was at least as large as that between the two categories in the original dataset was tallied. The frequency with which that occurred then corresponds to the P-value for the null hypothesis, that the two categories would stem from a common distribution. Within each experiment, these raw P-values were corrected for multiple tests (across the bins) using Bonferroni [30] correction. The categories were thus considered to be significantly different if the raw P-values were below 0.05/N, where N is the number of bins.

### Significance test for protein pair difference categories

To test whether the two homology types (orthology and paralogy) differed with regards to how common the different domain architecture similarity classes and domain swapping event types were, we performed a variant of Pearson's χ^2 ^test [31]. For each species comparison, the number of pairs falling into each category was tabulated (as columns, with the different classes/event types represented as rows). The χ^2 ^statistic was computed using a custom Perl script (available by request) for the goodness-of-fit relative to a distribution of points across difference or architecture change categories estimated from both pair types together. The resulting statistic was then compared to a χ^2 ^table of the respective number of degrees of freedom (f = [M - 1]×[N - 1] for M rows and N columns).

The χ^2 ^is not recommended if any cell has an expected count lower than 5. Because of this, the difference category NotSame and the architecture change categories domain shuffling and segment duplication/deletion were excluded from the respective analyses, making f = 2 in both cases. Even so, in the architecture change comparison, expected counts went below 5 for the prokaryote comparisons, meaning that the test cannot be considered reliable for those species.

## Results

### Characterizing protein pair types

**Figure 2:**
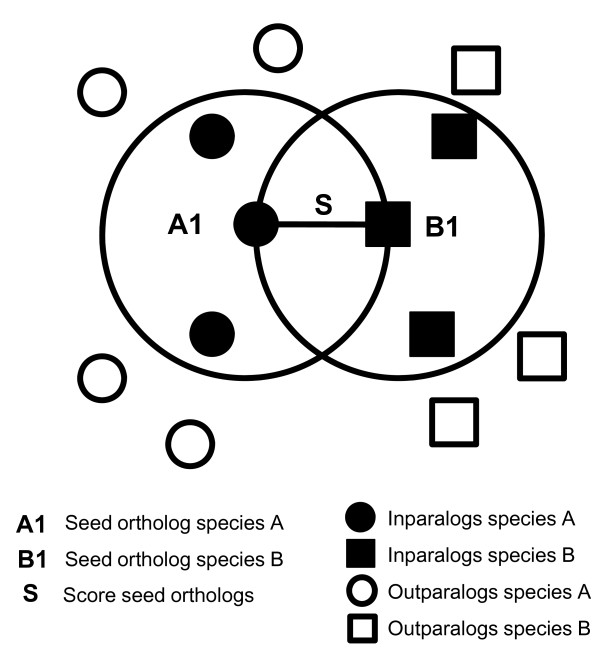
**Illustration of orthology definitions**. **Species **A and B are compared. Proteins A1 and B1 are each others' closest cross-species homologs and are considered seed orthologs. Other proteins in A and B are inferred to have descended from the same ancestral gene (and thus be inparalogs) if they are closer to the seed orthologs (inside the circles) than the seed orthologs are to each other. If this is not the case (proteins outside the circles), then they are inferred to be outparalogs. Two inparalogs in the same species (any two black circles or squares, respectively) form an inparalog-inparalog (iP) pair. Any cross-species pair of cluster members (any black circle vs any black square) form an ortholog-ortholog (O) pair. For all cluster members, the pair formed with the closest same-species protein outside the cluster (black square and closest white square, black circle and closest white circle) form an ortholog-closest same-species outparalog (oPs) pair. Likewise for all cluster members, the pair formed with the closest cross-species protein outside the cluster (black square and closest white circle, black circle and closest white square) form an ortholog-closest cross-species outparalog (oPx) pair.

We computed the levels of domain architecture and primary sequence conservation for pairs of orthologous proteins, and compared these with corresponding figures for paralogous proteins at the same evolutionary divergence. We considered sets of ortholog clusters as defined by InParanoid, between *Homo sapiens *and 40 other species. From these clusters, we extracted protein pairs falling into four types depending on their orthology status. See Figure 2 for an illustration of how the orthology status of a protein is defined. Ortholog (O) pairs contain one protein from *Homo sapiens *and one orthologous protein from another species. Inparalog (iP) pairs are pairs of proteins within either *Homo sapiens *or another species, which are part of the same ortholog cluster, and which thus have arisen through a gene duplication following the divergence of the two species. Outparalog pairs of two types were considered: Closest cross-species (oPx) and same-species (oPs) outparalogs. The former pairs consist of a protein from one species and the protein in the other species to which it is the most similar outside of the ortholog cluster. The latter, analogously, consist of a protein in one species and the most similar protein in the same species outside the cluster.

We would expect function to be more often conserved in O pairs than in other pair types because in other pair types, gene duplications would have relaxed pressure on one of the copies to retain the ancestral function. We further subdivided the O pair category into pairs from clusters with only a single member in each species (1-1 orthologs), and pairs where gene duplication has occurred in either species. In the non-duplicated case, function conservation should be more frequent than in the case with duplication, as the latter offers more opportunity for undergoing and retaining functional shifts (neo- [32] or subfunctionalization [33]).

### Absolute domain architecture conservation

We developed a method to score the degree of domain architecture conservation between two proteins, called the DA-score. This extends the DCS score of Song et al [20] by not only considering the domain content but also the actual alignment of domains (See Methods). This way the domain order is taken into account. In this, it is similar to the domain architecture distance used by Björklund et al [10], but inverted and normalized to form an actual similarity measure. As previously stated, while the method of Lin et al [18] also provides a similarity measure for domain architectures, it is less straightforward and optimized for detecting homology rather than for describing the degree of architectural differences between proteins.

To avoid biasing the results towards large clusters we calculated the average score for all protein pairs of a given pair category in each cluster first, and then calculated the average of these average cluster values for each category. This is equivalent to normalizing by cluster size, which makes sense as an approach to avoid biasing the results in favor of trends exhibited by only a few large clusters. As our analysis is pairwise, the presence of a small number of large clusters, either artifacts or consequences of intensive gene duplication within some gene families, could lead to a relatively small number of genes completely dominating the results of the analysis. This might hide general trends, as well as bias the analysis unfairly towards certain gene functions or families.

**Figure 3:**
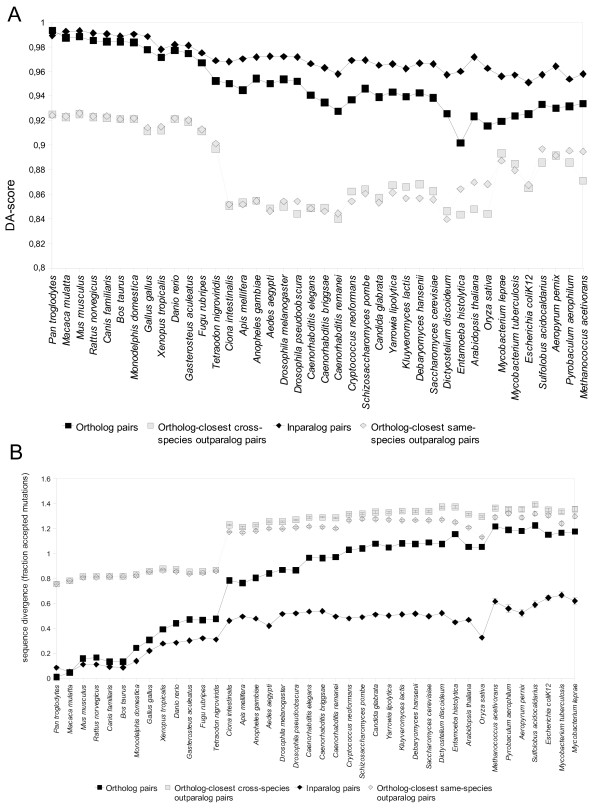
**A. Absolute domain architecture conservation of ortholog and paralog pairs**. The average DA-score for each pair type is shown for comparisons between human and other species, sorted by their distance to human. Inparalog pairs have diverged after the speciation of the two species and are therefore generally have the most conserved domain architecture. Ortholog pairs are considerably more conserved in domain architecture than the outparalogs. **B Sequence divergence of ortholog and paralog pairs**. The average number of expected substitutions per position for each protein pair type is shown for comparisons between human and other species, sorted by their distance to human. The average divergence increases relatively smoothly at first within the vertebrates but a jump is seen at the vertebrate-invertebrate border.

Figure 3A shows the domain architecture conservation for comparisons between *Homo sapiens *and successively more distant species (as defined by the NCBI Taxonomy) for the different pair types. Inparalog pairs, having diverged later than any of the other pair types, are in all cases the most conserved, and this difference becomes more pronounced the further back the speciation event happened. The graph shows a gradual drop in similarity across all pair types, except a striking jump for outparalog pairs at the vertebrate/invertebrate border. The point of this accelerated outparalog divergence supports the theory of whole-genome duplications at the vertebrate root [34].

As seen in Figure 3A, increasing evolutionary distance is related to an increase in architectural differences, although there is a type of plateau behavior since sequences cannot grow too different and still be recognized as homologs. It is noticeable that outparalog pairs to exhibit higher domain architecture conservation in prokaryotes than in plants. Possibly this is a consequence of prokaryotic proteins in general having fewer domains [35].

**Figure 4:**
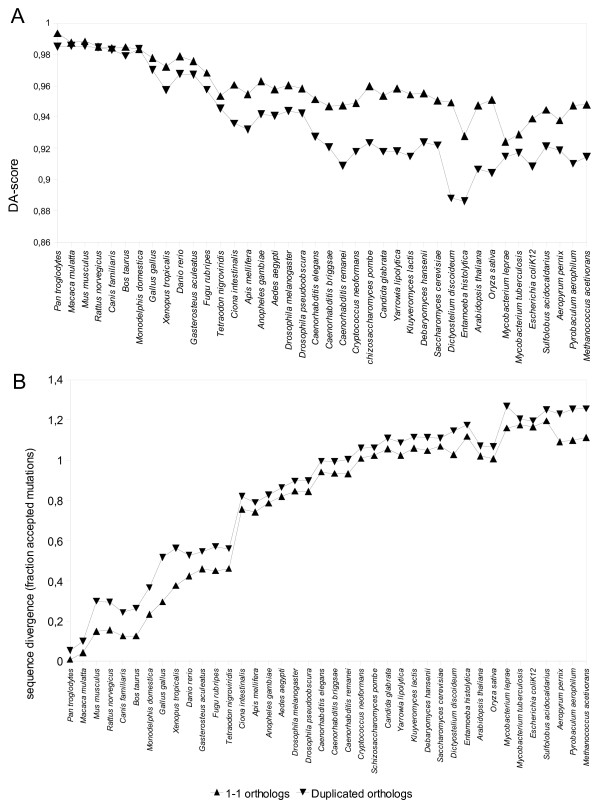
**A. Absolute domain architecture conservation of duplicated orthologs and 1-1 orthologs**. The average DA-score for each protein pair type is shown for comparisons between human and other species, sorted by their distance to human. Domain architecture is clearly more conserved in clusters where no gene duplication has taken place. The scores for each pair type are first averaged within each cluster so that each cluster contributes equally to the average scores regardless of size. **B. Sequence divergence of duplicated orthologs and 1-1 orthologs**. The average number of substitutions per position for each protein pair type is shown for comparisons between human and other species, sorted by their distance to human. The greatest divergence between the pair types is seen for comparisons of human vs non-primate vertebrates.

The analysis was also done for orthologs split into 1-1 orthologs and duplicated orthologs, as shown in Figure 4A. As expected, 1-1 orthologs have more conserved domain architecture. Supplementary Table 2 shows the fraction of these two pair types that have identical domain architectures. Notably, for 1-1 ortholog pairs, the fraction of pairs with identical architectures is on average 11.2% higher than for duplicated orthologs.

Figure 3B shows the mean sequence divergence for the same species comparisons. As a measurement of sequence divergence, we calculated the evolutionary distance as expected number of amino acid substitutions per position between each pair of proteins. Again, inparalog pairs were most similar, followed by ortholog pairs and paralogs. However, here the ortholog and paralog curves came much closer to each other in the more distant species. In other words, sequence divergence does not strongly distinguish orthologs from other homologs at high evolutionary distance. A clear jump also in sequence divergence is seen at the vertebrate/invertebrate border.

Figure 4B shows the mean sequence divergence for duplicated orthologs relative to 1-1 orthologs. For all species comparisons, the duplicated orthologs had on average diverged more than 1-1 orthologs. Interestingly, this gap is considerably wider for most vertebrates than for non-vertebrates. This may again be a consequence of large-scale neofunctionalization following whole-genome duplications at the root of the vertebrate lineage [34]. The fact that the gap is not larger for more distant vertebrates is harder to explain in this manner, and may instead hint at a generally higher degree of neofunctionalization following gene duplication within vertebrates.

While the overall trends in Figures 3, 4 are clear, neither the DA-score nor the sequence divergence changes perfectly smoothly as we move to more distant species comparisons. This is to be expected for several reasons. The NCBI taxonomy is not perfect, and species that all share the same last common ancestor with human cannot be internally ranked. Furthermore, evolutionary rates may vary between lineages, so that distance and branching order in a rooted topology will not always correspond perfectly.

### Domain architecture conservation relative to primary sequence conservation

Given that InParanoid assigns orthology status using relative BLAST [36] scores, it is not surprising that orthologs on average should exhibit lower sequence divergence than paralogs.

We therefore analysed how DA-score is affected by sequence divergence, and whether this effect is the same for protein pairs of different orthology status. Data from all species comparisons were pooled, and the clusters were divided into bins based on average sequence divergence for each pair type. This can be seen as way to normalize the DA-score with sequence divergence, to determine whether there are differences between orthologs and non-orthologs with respect to how well architecture is conserved as primary sequence diverges. Average DA-score was first calculated for all pair types within each cluster, and then these values were averaged across all clusters. The first step was done to avoid biasing the results towards large clusters.

Are differences between categories significant in a bin analysis of this type? Sparsely populated bins might yield spuriously large differences between the mean values for the two categories just by chance. To avoid this we performed a randomization test for a significant (Bonferroni corrected p < 0.05) difference between the category means within each bin.

**Figure 5:**
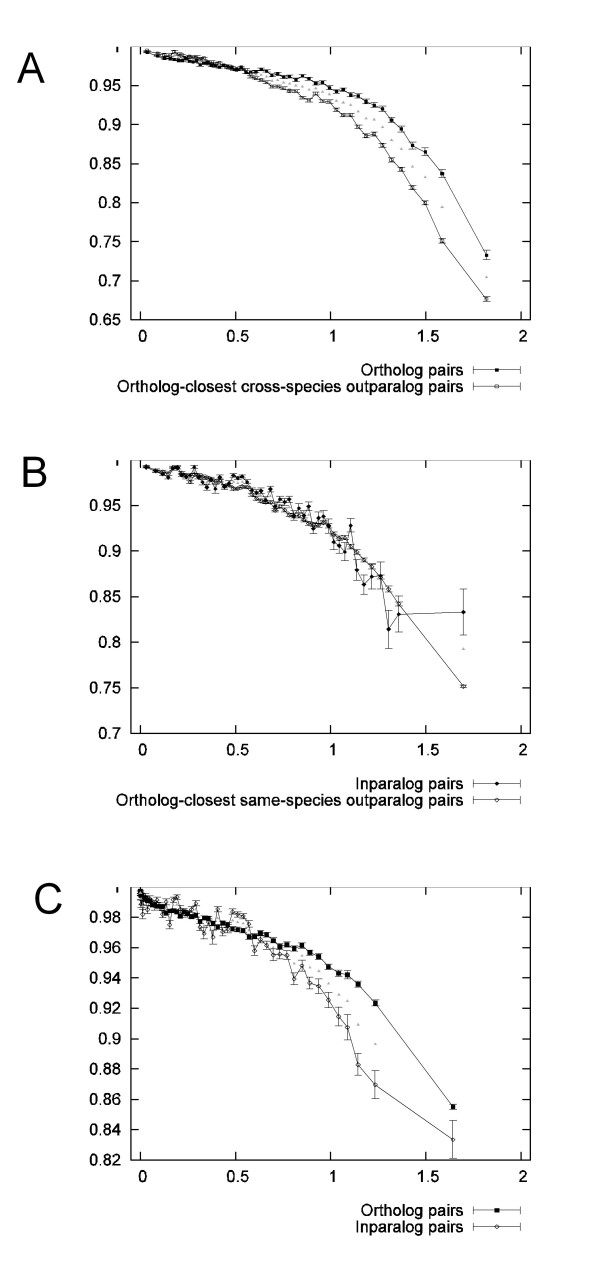
**A. Domain architecture conservation across all species for pairs of orthologs and closest cross-species outparalogs**. DA-score is averaged within ranges (bins) of sequence divergence. The scores for each pair category were first averaged within each cluster so that each cluster contributes equally to the average scores regardless of size. Ortholog pairs have greater domain architecture conservation than outparalog pairs of equivalent sequence divergence, at least when sequence divergence is high. Error bars show the standard error of the means for each pair category. The triangle markers indicate significant (Bonferroni corrected p < 0.05) difference between the category means within each bin. In these plots, the data was divided into 50 bins. **B. The equivalent analysis for pairs of inparalogs and same-species outparalogs**. Inparalog pairs in this context are proteins within the same species (human or a model organism) that diverged after the speciation event, whereas outparalog pairs are proteins within the same species that diverged before the speciation event. Both types of pairs are paralogs, and they behave similarly as sequence divergence increases. **C. The equivalent analysis for pairs of orthologs and inparalogs**. Just as seen in A, mean DA-score falls significantly more quickly for paralogs than for orthologs as sequence divergence increases.

Figure 5A compares the mean DA-scores of ortholog pairs and cross-species outparalog pairs at different levels of sequence divergence. For sequence divergences higher than ca 0.5 expected substitutions per site, we observe that orthologs have significantly higher mean DA-score, and that the difference increases with increasing sequence divergence. For shorter distances, a significant although very weak seemingly opposite trend can be observed. As a control, Figure 5B is an equivalent comparison of paralog versus paralog, here inparalog pairs and same-species outparalog pairs. While some bins with significantly different means exist in this comparison as well, they are much fewer, and there is no visible separation between the categories. Figure 5C compares ortholog pairs with inparalog pairs, and again the ortholog pairs exhibit significantly higher mean DA-score than the paralog pairs for most bins above about 0.7 expected substitutions per site. Supplementary Tables 3A-C show the exact bin borders, number of clusters in each bin, P-values, as well as mean sequence divergence and DA-scores.

Conceivably, an analysis of this type may yield errors if the categories have a significantly different distribution of data points (clusters in this case) within each bin with respect to the variable which is binned on. False positive trends resulting from such conditions should increase in proportion if bins are made broader, and diminish or disappear if bins are made narrower. To investigate this we repeated the analysis using 10, 20, and 100 bins, which all showed the same trend (see Additional file 2, Figure S1A-C, Additional file 3, Figure S2A-C, and Additional file 4, Figure S3A-C). This indicates that differences in sequence divergence distribution within individual bins cannot explain the significant differences in DA-score we see between the categories.

These results, which appear to hold for ortholog and paralog pairs above an evolutionary separation of about 0.5-0.7 expected substitutions per site, indicate that conservation of domain architecture and of primary amino acid sequence are semi-independent properties, in the sense that protein pairs at the same level of sequence conservation will often vary predictably in architecture conservation depending on their orthology status. We interpret this as a higher relative conservation of function for orthologous protein pairs, which in turn confers a higher relative conservation of domain architecture than for other homologs. This in turn provides support for the widespread assumption that the domain architecture of a protein is informative with regards to its function.

### Comparison with previous work

As stated previously, Lin et al [18] investigated KOGs [19] clusters and found that 81% of architectures in their dataset belonged to a single KOG only, and that 65% of the KOGs in their dataset contained only a single architecture. Additional file 1, Table S4 presents equivalent results for InParanoid clusters in our dataset. The overall trend across species appears to be similar. However, using InParanoid clusters, slightly fewer architectures (75% on average) are found only in a single cluster, and significantly more clusters (82% on average) contain only a single architecture. The differences between the study outcomes could stem either from our approach using Pfam clans and collapsing repeat/motif families, which was not used by Lin et al [18], or it might reflect differences between KOGs and InParanoid in that the former tends to merge clusters which are distinct in the latter [37].

### Degree of architectural similarity

In order to analyse the nature of architecture differences between proteins, we defined four basic classes of domain architecture similarity. Protein pairs with identical domain architectures belong to class Identical. Protein pairs with the same domain set but without all domains perfectly aligned (that is, pairs having a different order and possibly a different number of domains of each type) belong to class SameContentNotAligned. Protein pairs with overlapping but not identical domain sets belong to class DiffContent, and protein pairs with disjoint domain sets belong to class NoShared.

**Figure 6:**
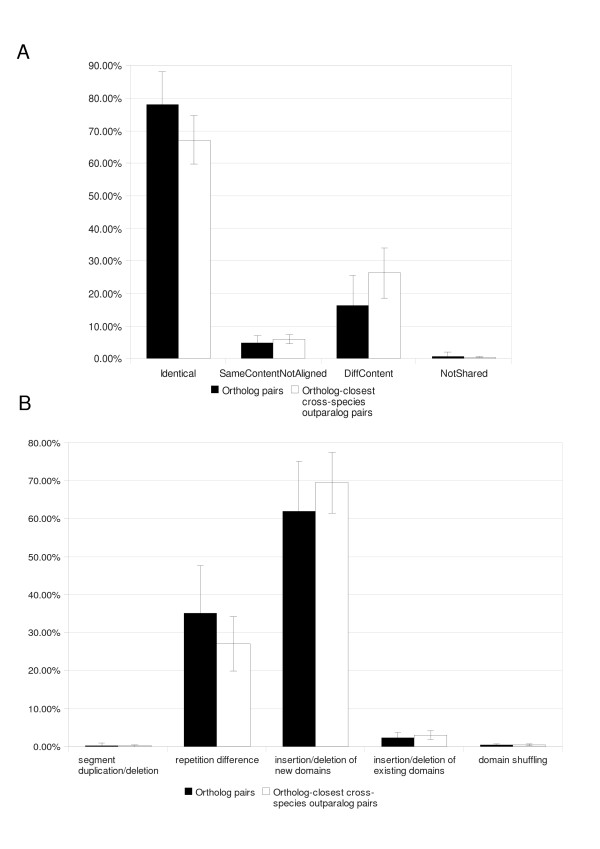
**A. Distribution of protein pairs across domain architecture similarity classes**. All ortholog and cross-species paralog pairs in each species were divided into four qualitatively distinct classes (See Results), that are plotted as averaged fractions over all species comparisons. **B. Distribution of protein pairs across domain swapping event categories**. Protein pairs with partially preserved domain architecture (classes SameContentNotAligned and DiffContent in A) were further characterised in terms of inferred domain swapping events according to the five categories defined in Figure 1. The chart shows averages over all species comparisons. Error bars indicate the standard deviation across species.

We compared how the ortholog (O) and cross-species outparalog (oPx) pairs fell into these classes. The overall trend across all species is shown in Figure 6A. O pairs generally have more identical domain architectures (true in 38 of 40 species) while oPx pairs more often have different domain content (true in 39 of 40 species). It is worth noting that it is very rare for two proteins to have the same domain content but not have identical domain architecture (SameContentNotAligned). In fact, this is substantially less frequent than having different domain content. Apparently, when functional divergence is allowed, this normally happens by also changing the domain content rather than merely shuffling the existing domains.

The numbers for individual species comparisons are listed in Supplementary Table 5A. We did a statistical test (χ^2^) to assess the whether the distribution across the three classes Identical, SameContentNotAligned and DiffContent (the number of pairs in the NotShared class were often too small for the χ^2 ^test to be suitable) was significantly different between O and oPx pairs. For 34 of the 40 species, the distributions were found to be significantly different (P < 0.05). All of the non-significant results were in prokaryotes.

### Characterizing domain swapping events

For protein pairs in the classes SameContentNotAligned and DiffContent (i.e., those proteins that share at least one domain but do not have identical architectures), we further analysed the presence of the five different types of domain swapping events described in Figure 1 (segment duplication/deletion, repetition difference, insertion/deletion of new domains, insertion/deletion of existing domains, domain shuffling).

The overall averaged results are shown in Figure 6B, and the per-species details in Additional file 1, Table S5B. Again, we compare the distributions among these categories for O versus oPx pairs. For 35 of the 40 species, the distributions among the categories repetition differences, insertion/deletion of new domains and insertion/deletion of existing domains were found to be significantly different (P < 0.05) under a χ^2 ^test. (The numbers of pairs in the other two categories were too small for the test to be suitable). The non-significant species were all prokaryotes. Additionally, for two prokaryotes where the difference was significant, the applicability of the χ^2 ^test is questionable because at least one expected cell count was less than 5. Mainly, this difference seems to occur in oPx pairs having fewer repetition differences than O pairs (true in 35 of 40 species) and more often undergoing insertion/deletion of new domains (true in 34 of 40 species).

For both pair types, it is striking how rarely segment duplication/deletion, domain shuffling, and insertion/deletion of existing domains is observed. Almost all architectural differences can be explained by repetition differences or insertion/deletion of new domains. The fact that oPx pairs have a higher degree of insertion/deletion of new domains supports their generally more relaxed functional constraints.

### Position bias of domain architecture change events

As previous work [10,13] has shown that domain architecture changes preferentially seem to occur (or be fixated) at protein termini, we investigated whether such a trend holds true for this dataset as well. The change events within each orthology cluster were tallied, and the ratio of each position was taken for each cluster, then averaged across all clusters. Additional file 1, Table S6 shows the distribution of events across the N-terminal, middle, and C-terminal categories. The results are basically in agreement with previous studies [10,13]: terminal events are more common than architecture changes in the middle of proteins, with some bias towards the N-terminal end. The same pattern held across distributions between different pair types.

## Discussion

We defined a measure for domain architecture conservation, the DA-score, which together with our use of Pfam clans for increased sensitivity and collapsing of repeat/motif-type families, allowed us to compare degrees of architecture conservation in a functionally and evolutionarily sensible way. By taking the DA-score for InParanoid-defined orthologs, we were able to study in detail the degree of domain architecture conservation and variance exhibited by orthologous and other sequence pairs across a wide variety of species.

The central observation we make is that orthologs diverge more slowly in domain architecture than paralogs, relative to their divergence in primary sequence. This is consistent with higher selective pressure on orthologs to retain their ancestral domain architecture, and that orthologs are relatively more conserved with respect to function given that the particular domain architecture of a protein is required for its function to be carried out.

These conclusions are clear from the data for protein pair comparisons involving more distantly separated homologs, but not for close homologs. Similar observations were made by Peterson et al. [38] who reported higher structural similarity between orthologous domains than between paralogous domains at equivalent evolutionary separation, but only throughout the range of 30-70% sequence identity. Since no other strong and significant trends are present in the graphs, we view the strikingly higher relative conservation of orthologous architectures for distantly separated homologs as the most notable observation. Why do we not observe it for shorter sequence separation ranges? Some form of methodological or dataset artifact cannot be ruled out, but it could also correspond to a genuine biological difference between processes taking place over different timescales.

Kondrashov et al. [39] suggested, on the basis of a large-scale analysis of evolutionary rates in recent paralogs, that recently duplicated genes may in fact, despite seeming redundant, contribute to fitness through dosage effects. Under this model, this fitness benefit would maintain a duplicated paralog in the population under weak purifying selection. Once it has diverged sufficiently, mutations shifting its overall function may take place to fix it. If such functional shifts are associated with changes in domain architecture, our observations would match this model well. Initially, duplicated paralogs resist domain-architecture changing mutations as retaining the ancestral function still provides increased relative fitness. As time goes by, sequence changes accumulate such that eventually an architecture changing mutation results in a novel protein variant which is more, or at least not less, favored by selection, resulting in two distinct stages of paralog, but not ortholog, evolution.

We observe that orthologous sequences detected using InParanoid share most if not all of their domains. What are the functional implications of a domain architecture alteration between orthologs? InParanoid, just like all other ortholog predictors, uses evolutionary criteria to assign orthology, and no functional information. The definition of orthology by Fitch [1] is also purely based on evolutionary relationships, and does not require that orthologs have exactly the same function. It may thus be the case that a fraction of the orthologs detected by InParanoid have undergone functional changes, and this fraction is likely to coincide with the orthologs altered in domain architecture. Unfortunately, the present functional annotation is too coarse to assess whether this is indeed the case.

Some insight into this issue can be provided by looking at the distributions of DA-scores for different pair types. In Figure 4 the average DA-scores are shown, but the distribution in each sequence identity bin is relatively complex and difficult to represent well using standard measures. The majority of pairs for most bins and pair types have identical domain architectures (DA-score = 1.0). This fraction is higher for orthologs, especially non-duplicated ones, than for paralogs. For the pairs with a DA-score < 1.0, the trend across pair types is mostly reversed (see Additional file 5, Figure S4A); i.e. the mean DA-score of non-identical orthologs is lower than that of non-identical paralogs. It is not immediately obvious how this surprising observation should be interpreted. This pattern would be consistent with a scenario where function conservation (the normal situation between orthologs) dictates complete domain architecture preservation, yet when the function starts to diverge, the domain architecture does so too. For paralogs this dichotomy between identical/non-identical architectures would be less pronounced, as the requirements for function conservation are weaker.

One concern about the results presented here, is that InParanoid, like other schemes for systematically finding orthology relationships, is based on degrees of similarity between sequences, which might change as the domain architectures do. As long as InParanoid assignments are correctly made, there should be no problem. However, errors in cluster assignments, to the extent that they occur, are more likely to produce ortholog pairs with similar architectures and paralog pairs with dissimilar architectures, than the reverse. This risk for bias applies equally to any sequence- or structure-based method for orthology assignment, which includes all methods that could conceivably be used for a large-scale analysis of domain architecture evolution under orthology versus paralogy. In the absence of ways to evaluate the extent to which this factor impacts the result, it should be noted as a potential error source. However, to the extent that InParanoid itself is reliable, it should not strongly affect the outcome of this study.

We noted that both average sequence divergence and domain architecture conservation change noticeably as we cross the vertebrate-invertebrate border. This might be interpreted either as a consequence of high evolutionary rates early in the vertebrate lineage, a bottleneck effect where most sibling species of the early invertebrates did not survive, or an artifactual bias in the set of fully sequenced genomes available at this time. Another interesting option might be rapid evolution following two consecutive whole-genome duplication events, which have been inferred to have taken place early in the vertebrate lineage [34]. Given that we consider as many as 40 species, we feel confident that this phenomenon is biologically relevant rather than an artifact of the dataset.

## Conclusions

This study has employed large scale data analysis to investigate the hypothesis that orthologs have a higher level of domain architecture conservation relative to their evolutionary divergence than other homologs. Strong support for this proposition was found, at least for protein pairs that have diverged beyond a certain point. We conclude that it is possible to infer that orthology is accompanied by a selective pressure to retain domain architecture, and that the specific order of domains seems to be important to the function carried out by a set of orthologous proteins. Further work should address the lack of this trend for recently diverged homologs.

## Authors' contributions

ES conceived the study and developed the DA-score together with IP. IP wrote most of the underlying software and performed some initial analysis. KF introduced the sequence divergence compensation procedure, carried out the main analysis, and performed all statistical tests. KF and ES wrote the manuscript. All authors read and approved the final manuscript.

## Supplementary Material

Additional file 1 **Supplementary tables**. This file contains the following tables: *Table S1: *This table shows the full selection of species, as well as how many clusters and proteins were included in each species comparison analysis. *Table S2: *This table shows the fraction of ortholog pairs from clusters with and without duplications, respectively, that have identical domain architectures. *Table S3: *Bin borders, averages, number of clusters falling into each bin, and permutation test P values underlying Figure 5A-C. Note that these P-values should be measured against a Bonferroni-corrected threshold, so for p < 0.05 and N = 10 samples, significance requires that the value in the cell is smaller than 0.005. *Table S4: *Spread of distinct architectures across clusters. *Table S5: ***A**. Distribution of protein pairs across domain architecture similarity classes in the various species comparisons. **B**. Distribution of protein pairs where architectures differed across domain architecture change event classes in the various species comparisons. *Table S6: *This table shows the distribution of domain architecture change events across the N-terminal, middle, and C-terminal domain position categories. Click here for file

Additional file 2 **Supplementary Figure S1A-C**. This file contains the following figure: *Figure S1*. **A**. Domain architecture conservation across all species averaged within ranges (bins) of sequence divergence, for pairs of orthologs versus closest cross-species outparalogs. The scores for each pair category were first averaged within each cluster so that each cluster contributes equally to the average scores regardless of size. Error bars show the standard error of the means for each pair category. The triangle markers indicate significant difference between the category means within each bin. In these plots, the data was divided into 10 bins. **B**. The same analysis for inparalogs versus same-species outparalogs. **C**. The same analysis for orthologs versus inparalogs. Click here for file

Additional file 3 **Supplementary Figure S2A-C**. This file contains the following figure: *Figure S2A-C*. Same as S Additional file 2, Figure S1A-C but with 20 bins. Click here for file

Additional file 4 **Supplementary Figure S3A-C**. This file contains the following figure: *Figure S3A-C*. Same as Additional file 2, Figure S1A-C but with 100 bins. Click here for file

Additional file 5 **Supplementary Figure S4A-C**. This file contains the following figure: *Figure S4A-C*. Same as Figure 5A-C but excluding clusters where all pairs have a DA-score of 1.0, to specifically consider clusters where architectures are not perfectly conserved. Click here for file
