# Supplementary figures and images for: Domain architecture conservation in orthologs
Source: BMC Bioinformatics. 2011 Aug 5;12:326. doi: 10.1186/1471-2105-12-326 (PMC3215765; doi:10.1186/1471-2105-12-326)

## Slide 1
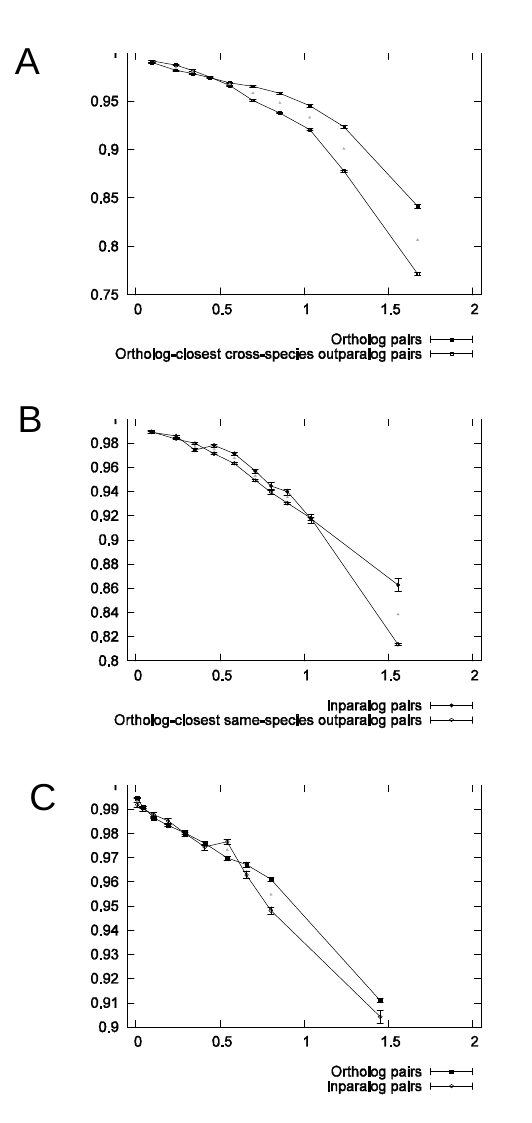

A
B
C

Supplement: Additional file 2 — Supplementary Figure S1A-C. This file contains the following figure: Figure S1. A. Domain architecture conservation across all species averaged within ranges (bins) of sequence divergence, for pairs of orthologs versus closest cross-species outparalogs. The scores for each pair category were first averaged within each cluster so that each cluster contributes equally to the average scores regardless of size. Error bars show the standard error of the means for each pair category. The triangle markers indicate significant difference between the category means within each bin. In these plots, the data was divided into 10 bins. B. The same analysis for inparalogs versus same-species outparalogs. C. The same analysis for orthologs versus inparalogs. [file 1471-2105-12-326-S2.PPT]

## Slide 1
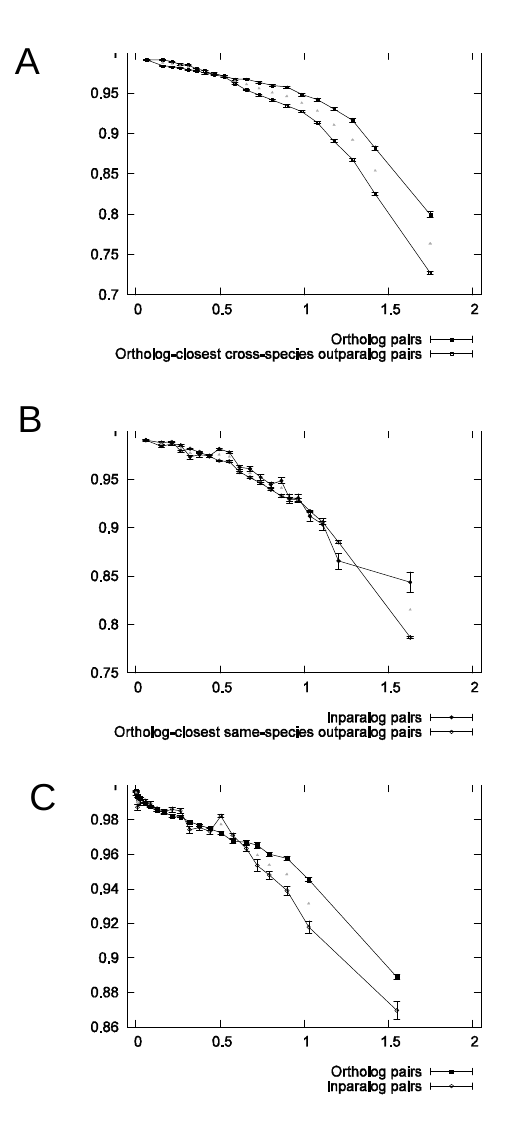

A
B
C

Supplement: Additional file 3 — Supplementary Figure S2A-C. This file contains the following figure: Figure S2A-C. Same as S Additional file 2, Figure S1A-C but with 20 bins. [file 1471-2105-12-326-S3.PPT]

## Slide 1
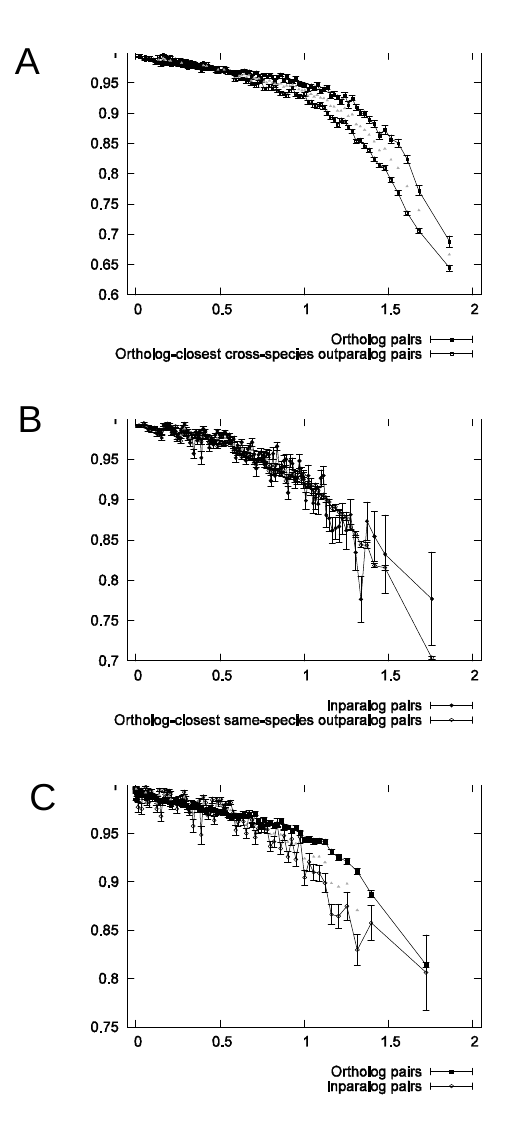

A
B
C

Supplement: Additional file 4 — Supplementary Figure S3A-C. This file contains the following figure: Figure S3A-C. Same as Additional file 2, Figure S1A-C but with 100 bins. [file 1471-2105-12-326-S4.PPT]

## Slide 1
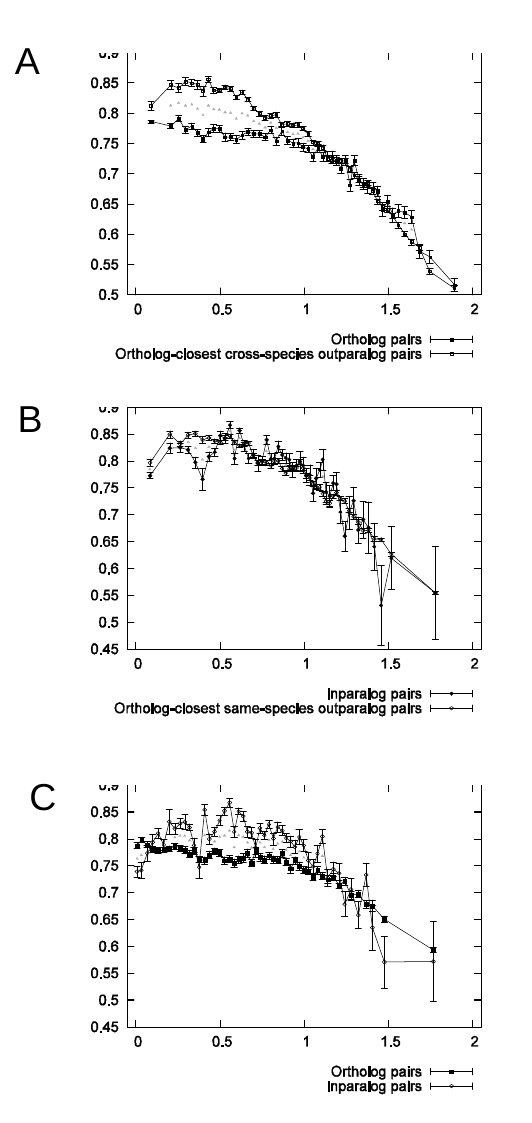

A
B
C

Supplement: Additional file 5 — Supplementary Figure S4A-C. This file contains the following figure: Figure S4A-C. Same as Figure 5A-C but excluding clusters where all pairs have a DA-score of 1.0, to specifically consider clusters where architectures are not perfectly conserved. [file 1471-2105-12-326-S5.PPT]
